# Lysozyme-Imprinted Surface Plasmon Resonance Chips Decorated with Gold Nanoparticles for Lysozyme Detection

**DOI:** 10.1021/acsomega.5c01607

**Published:** 2025-06-26

**Authors:** Şeyma Eriş, Duygu Çimen, Adil Denizli

**Affiliations:** a Department of Chemistry, 37515 Hacettepe University, Ankara 06800, Turkey; b Bioengineering Division, 37515 Hacettepe University, Ankara 06800, Turkey

## Abstract

In this study, lysozyme-imprinted (Lyz-AuNP-MIP) and nonimprinted (AuNP-NIP) surface plasmon resonance (SPR) sensors modified with gold nanoparticles (AuNPs) were prepared for signal amplification and real-time determination of lysozymes from both aqueous lysozymes and artificial plasma, artificial urine, and artificial tear solutions. Furthermore, lysozyme-imprinted (Lyz-MIP) SPR sensors without AuNPs were prepared to study the signal enhancing effect of the AuNPs. The surface morphology of all prepared SPR sensors was characterized by atomic force microscopy and contact angle measurements. After the characterization study, kinetic studies were carried out using lysozyme solutions (pH 7.4 phosphate buffer), artificial plasma, artificial urine, and artificial tear prepared from 0.01–500 μg/mL concentrations using Lyz-AuNP-MIP SPR sensors for lysozyme determination. Using the kinetic analysis data obtained from the Lyz-AuNP-MIP SPR sensors, the limit of detection and limit of quantification were 0.008 and 0.026 μg/mL, respectively. Competitive agents, such as myoglobin and hemoglobin, were used to demonstrate the selectivity of Lyz-AuNP-MIP and AuNP-NIP SPR sensors. Furthermore, after optimizing the experimental studies for lysozyme determination, lysozyme determination was also performed in artificial plasma, artificial urine, and artificial tear solutions, and the recoveries were calculated to be approximately 99%.

## Graphical abstract

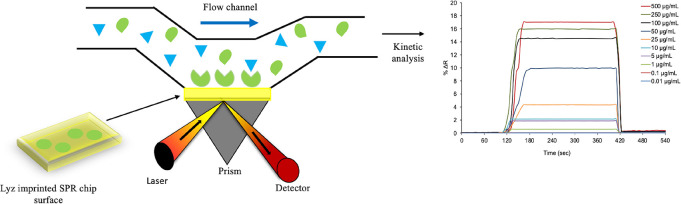

## Introduction

Lyzozyme (Lyz), a natural antimicrobial defense mechanism in the body, acts by breaking down the cell walls of microorganisms and is found in various tissues. Due to this property, it is considered an important component that protects the body from a range of pathogenic microorganisms. The detection of lysozyme is crucial for the diagnosis of certain diseases such as Alzheimer’s and Crohn’s. Lysozyme, also called muramidase, is widely found in various organisms such as bacteria, bacteriophages, fungi, plants, and mammals.^1^ It is one of the proteins known for its activity against Gram-positive bacteria. It breaks the glycosidic bonds between *N*-acetyl muramic acid and *N*-acetyl glucosamine in the peptidoglycan cell wall of bacteria. It is considered as the body’s own antibiotic since it breaks down bacterial cell walls. It is also of great importance in protein and enzyme studies.^2^ Lysozyme is found in many body fluids and tissues such as saliva, tears, urine, and spleen. Lysozyme, which is also found in human milk, is passed on to children through breastfeeding and helps in the formation of their immune system.^3^ Lysozyme has a molecular weight of 129 amino acids and is approximately 14–15 kDa. It has an isoelectric point of approximately pH 11.^4^ Lysozyme is found in human milk and saliva in healthy individuals at approximately 21.4 and 7 μg/mL lysozyme, respectively, while patients suffering from leukemia and kidney disease have more than 15 or even 100 μg/mL lysozyme. Furthermore, lysozyme concentrations are lower in urine and serum samples from normal human adults (approximately 0.18 and 1.7 μg/mL, respectively).^5^ It is used as a biomarker in the diagnosis of various diseases such as Alzheimer’s, breast cancer, and rheumatoid arthritis.^6^ Increased lysozyme levels are also nonspecific indicators for diseases such as leukemia and meningitis. High levels of lysozyme may indicate infection in the wound fluid, bacterial meningitis in the cerebrospinal fluid, and leukemia in urine.^7^

Analytical devices such as sensors contain a combination of simple and convenient converter receptors that contain biological recognition elements. SPR is a technique that uses a thin metal film to reflect light and is based on the intensity of the reflected light in a prism. The reason why SPR sensors are popular for biomolecule detection is their simplicity, high specificity and sensitivity, real-time measurement, low cost, and no need for labeling. SPR, an optical technique, is used to measure the refractive index changes when the target molecule is bound or absorbed to the sensor surface. In recent years, SPR sensors have become commonly employed for detecting various biomolecules because of their simplicity, specificity, and sensitivity. Real-time measurements, no need for labeling, and easy miniaturization have led to their widespread use. The amount of analyte and the response units obtained from shifting angles can be monitored in real time.^8–13^

In recent years, noble-metal-based nanoparticles, especially gold and silver, are frequently preferred in the preparation of sensors because of their ability to enhance the sensitivity of sensors due to their ability to enhance responses. Gold nanoparticles (AuNPs) are used in the preparation of sensor surfaces due to their properties such as large surface areas, good conductivity, strong adsorption ability, and biocompatibility. AuNPs and MIPs are prepared with different approaches such as direct electrostatic unification, polymer capture, covalent binding, and electroplating methods.^14,15^ The molecular imprinting technique is a method that aims to produce molecularly imprinted polymers (MIPs) with special binding sites that can properly complement a certain shape, size, and functional groups. The basic principle of this technique is the creation of a polymer material that interacts with a target molecule and then forms a template of this molecule. The molecular imprinting method is the process of polymerizing the target molecule together with a suitable monomer and cross-linker and is an important method because it creates specific recognition and binding sites for the target molecule. After polymerization, the target molecule is removed from the polymer structure with the help of a suitable solvent, and cavities specific to the target molecule are formed in the polymer structure. As a result, there are special binding sites with high selectivity specific to the target molecule in terms of shape, size, and functionality on the polymer surface. The most important advantages of these polymers are their high selectivity and affinity for the target molecule used in the imprinting method, stable chemical, physical, and mechanical properties, resistance to high temperature and pressure, easy and cheap synthesis, long shelf life, and reusability.^16,17^

In this study, we aimed to prepare real-time and highly selective lysozyme-imprinted and nonimprinted SPR sensors for the determination of lysozyme. AuNPs were used in the preparation of lysozyme-imprinted and nonimprinted SPR sensors to enhance the sensitivity of the SPR sensor signal response. In addition, the lysozyme-imprinted SPR sensor was also prepared without AuNPs. First, the surface characterization of the lysozyme-imprinted and nonimprinted SPR sensors was performed by atomic force microscopy and contact angle measurements. Aqueous lysozyme solutions (0.01–500 μg/mL) prepared in different lysozyme concentration ranges were subjected to kinetic analysis, and association kinetic analysis, equilibrium analysis, and adsorption isotherm models were investigated. Furthermore, the prepared SPR sensors were comprehensively evaluated by selectivity and reusability analyses. Finally, kinetic analyses were performed with artificial plasma, artificial urine, and artificial tear samples to demonstrate the applicability of the SPR sensor in complex samples.

## Experimental Section

### Materials

Lysozyme as a template molecule was from Fluka. Ethylene glycol dimethacrylate (EGDMA), 2-hydroxyethyl methacrylate (HEMA), azoisobisbutyronitrile (AIBN) and allyl mercaptan, artificial tears (Bausch & Lomb, Rochester, USA), and artificial plasma were purchased from Sigma-Aldrich, USA. SPR sensor chips were purchased from Genoptics. All other chemicals of analytical grade were purchased from Merck.

### Preparation of Lysozyme-Imprinted and Nonimprinted SPR Sensors

Before lysozyme imprinting on the surface of the SPR chip, the SPR chip surface was washed with piranha solution prepared at a ratio of 3:1 v/v H_2_SO_4_:H_2_O_2_ in order to get rid of the organics bound to the surface. Then, it was washed with ethyl alcohol and rinsed with deionized water. It was left to dry in an oven set at 40 °C. After this process, the surface was made suitable for organic binding and the binding process of thiol groups was carried out. Allyl mercaptan was used to form the thiol group (−SH).

An *N*-methacryloyl-L-tryptophan (MATrp) monomer was used as a functional monomer in the preparation of lysozyme-imprinted (Lyz-AuNP-MIP) and nonimprinted (AuNP-NIP) SPR sensors. For the preparation of a lysozyme (Lyz)-imprinted SPR chip, first, 1 mmol of Lyz and 2 mmol of the MATrp complex were formed and the prepolymerization mixture was mixed in a rotator at 20 rpm for 30 min. Gold nanoparticles (AuNPs) were synthesized by reducing HAuCl_4_ salt to Au using sodium citrate by the Turkevich method as in a previous study.^14^ Then, 2 mmol AuNPs were added to the Lyz:MATrp prepolymerization complex and the template molecule was mixed at 20 rpm for 30 min to ensure coordination between the monomer and AuNPs. To this mixture were added 1 mmol of the HEMA monomer and 2 mmol of EGDMA as a cross-linker. Two mg of AIBN was added to this prepared polymerization mixture as an initiator. This polymerization mixture was dropped into the SPR chip surface functionalized with thiol groups as 4 μL, and a homogeneous distribution was achieved on the chip surface using a spin coater (LAURELL, WS 650Mz-23NPP, USA). A polymerization reaction was carried out under a UV lamp (365 nm, 100 W) for 30 min to form a polymeric film on the SPR chip surface (Figure 1). The nonimprinted AuNP-NIP SPR sensor was prepared in the same way using the Lyz-AuNP-MIP SPR sensor preparation procedure steps without using lysozyme as the template molecule. As a final step, lysozyme was removed from the SPR sensor surface with a 10% (v/v) ethylene glycol solution. The desorbed SPR sensor surface was washed with a deionized water:ethyl alcohol mixture for 1 h and dried in an oven. In order to examine the impact of the involvement of AuNPs to increase the SPR signal response, a lysozyme-imprinted polymeric film-based SPR sensor prepared without AuNPs was prepared as a control experiment.

**Figure 1:**
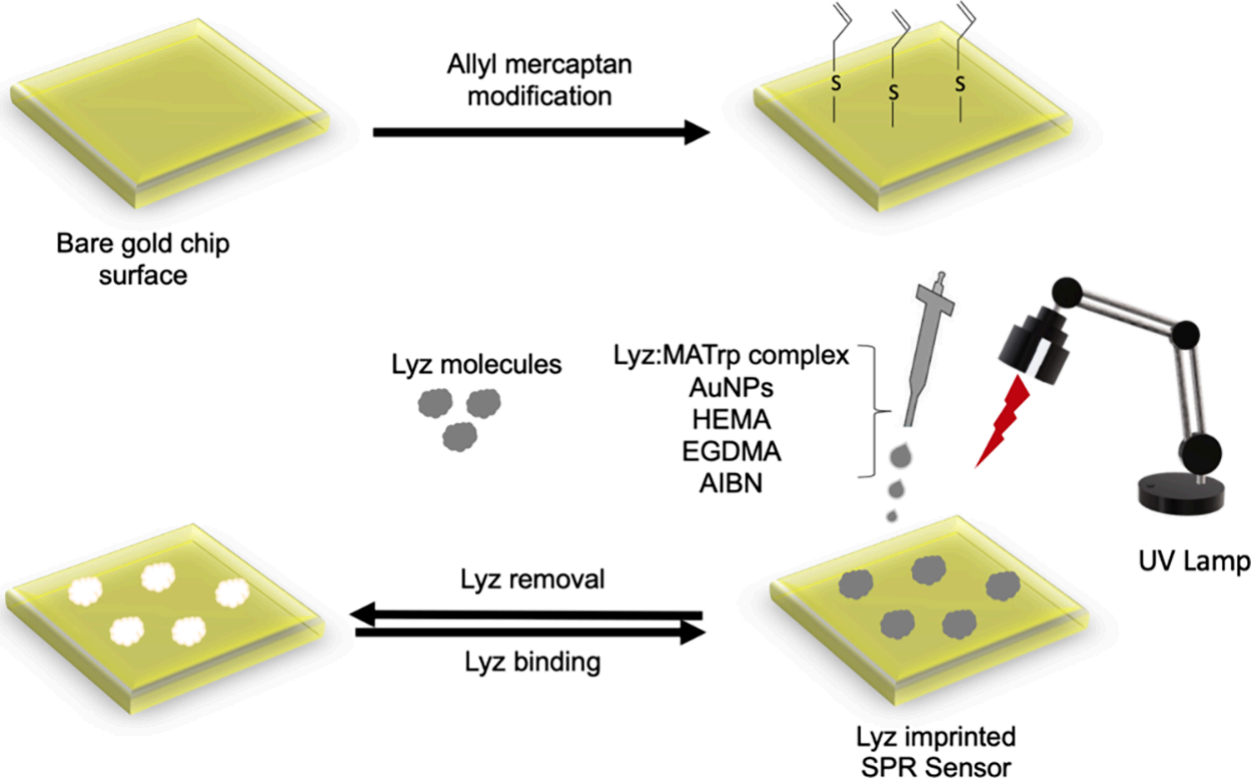
Schematic representation of the process of making lysozyme-imprinted SPR sensors.

For the preparation of lysozyme-imprinted (Lyz-MIP) SPR sensors, first, the Lyz:MATrp prepolymerization mixture consisting of 1 mmol of Lyz and 2 mmol of the MATrp monomer was prepared by mixing them in a rotator at 20 rpm for 30 min. One mmol of the HEMA monomer and 2 mmol of EGDMA as a cross-linker were added to the prepared prepolymerization mixture. Two mg of AIBN was added to this polymerization mixture as an initiator. Four μL of this prepared mixture was dropped onto the SPR chip surface modified with thiol groups, and a spin coater (LAURELL, WS 650Mz-23NPP, USA) was used to ensure homogenization on the chip surface. The polymerization reaction was carried out under a UV lamp (365 nm, 100 W) for 30 min to form a polymeric film on the SPR chip surface. As the last step, lysozyme was removed from the SPR sensor surface with a 10% (v/v) ethylene glycol solution. The desorbed SPR sensor surface was washed with a deionized water:ethyl alcohol mixture for 1 h and dried in an oven.

### Characterization Studies

#### Characterization of AuNPs

The characterization of AuNPs was performed by transmission electron microscopy (TEM) and a Nano Zetasizer Instrument (NanoS, Malvern Instruments). The morphology of AuNPs was characterized in detail by using transmission electron microscopy (TEM). For this purpose, AuNPs were first placed on a copper-coated grid and then dried at room temperature. TEM micrographs were taken using a high-resolution TEM microscope, FEI Tecnai G2 F30 model, at 200 kV. This process was performed to determine critical properties of the nanoparticles such as size, shape, and distribution. The dynamic light scattering (DLS) analysis was performed using a Nano Zetasizer instrument (NanoS, Malvern Instruments) to determine the hydrodynamic size of the AuNPs. In DLS analysis, the density of deionized water was set at 0.88 mPa s with a refractive index of 1.33′. Then, the solution of AuNPs was placed in the sample chamber of the analyzer and size analysis was performed. The size analysis measurements for AuNPs in a quartz cuvette with 1.0 mL nanoparticle solution were performed three times repeatedly with a Zetasizer. The absorbance of AuNPs was measured with a UV–vis spectrophotometer (SHIMADZU UV-1601 model, Tokyo, Japan) in the wavelength range 200–700 nm.

#### Characterization of SPR Sensors

Surface characterization of the unmodified SPR chip, Lyz-AuNP-MIP, AuNP-NIP, and Lyz-MIP SPR sensors was performed by atomic force microscopy (AFM) and contact angle studies. The roughness of unmodified SPR chip, Lyz-AuNP-MIP, AuNP-NIP, and Lyz-MIP SPR sensor surfaces was carried out using an atomic force microscope manufactured by NanoMagnetics Instruments. SPR chips were fixed to the sample holder with double-sided carbon tape, and imaging studies were carried out in air with an oscillation resonance frequency of 341.30 kHz and half mode. Vibration and null vibration amplitudes were 1 and 2 V_RMS_, respectively. Samples were images obtained from an area of 2 × 2 μm^2^ for the SPR chip surfaces at a scanning speed of 2 μm/s and a resolution of 256 × 256 pixels. Contact angle measurements of the SPR sensor surfaces were analyzed by using KRUSS DSA100 (Hamburg, Germany). Hydrophilicity properties were determined by dropping water onto the surfaces of the SPR sensors by using the sessile drop method. Water was dropped on different areas of the chip surface, and detailed photographs were taken for each area. These photographs were used to determine the contact angle of each area. The contact angle values determined for the unmodified SPR chip surface and the Lyz-AuNP-MIP, Lyz-MIP, and AuNP-NIP SPR sensor surfaces were determined using DSA2 software and calculated by taking the average of 10 separate measurements. The contact angle values were determined by using DSA2 software during the analysis process.

### Kinetic Studies of SPR Sensors

Kinetic analyses for the determination of lysozyme were performed on an SPRimager II (GWC Technologies, WI, USA) with Lyz-AuNP-MIP, AuNP-NIP, and Lyz-MIP SPR sensors. In this study, kinetic analyses were performed at room temperature, using a wavelength of 800 nm and prism material (SF10 glass). In order to determine lysozyme, kinetic analyses were performed on lysozyme aqueous solutions at a concentration of 0.01–500 μg/mL prepared in pH 7.4 phosphate buffer. First, before the kinetic analyses were started, 1.0 M pH 7.4 phosphate buffer was passed over the SPR sensor surface at a flow rate of 2 mL/min for 2 min. Then, lysozyme solutions at different concentrations between 0.01 and 500 μg/mL were prepared and run on the SPR system at a flow rate of 2 mL/min for 5 min. Finally, kinetic analyses were completed by passing (10%) the ethylene glycol desorption solution through the SPR system for 2 min. In all kinetic analyses consisting of equilibrium adsorption–desorption steps, all steps including the total analysis time were performed in approximately 9 min. The obtained SPR signals were monitored in real time on the SPR system, and the obtained sensorgram results were evaluated by calculating the change in the refractive index of light versus time (% Δ*R*). The equations below were used to determine the equilibrium and kinetic isotherm parameters of the Lyz-AuNP-MIP SPR sensor.

$$\mathrm{Association}\;\mathrm{kinetic}\;\mathrm{analysis}:\;d\Delta R/dt=k_{a}C(\Delta R_{\max}-\Delta R)-k_{d}\Delta R$$ 1

$$\mathrm{Equilibrium}\;\mathrm{analysis}\;(\mathrm{Scatchard}):\;\Delta R_{\mathrm{ex}}/C=K_{A}(\Delta R_{\max}-\Delta R_{\mathrm{eq}})$$ 2

$$\mathrm{Langmuir}:\;\Delta R=(\Delta R_{\max}C/K_{D}+C)$$ 3

$$\mathrm{Freundlich}:\;\Delta R=\Delta R_{\max}C^{1/n}$$ 4

$$\mathrm{Langmuir}-\mathrm{Freundlich}:\;\Delta R=((\Delta R_{\max}C^{1/n}/K_{D})+C^{1/n})$$ 5

By measuring signal induced by Lyz bonding, the SPR sensor system’s response is measured as Δ*R*. The concentration of Lyz is *C*, and the equilibrium constants for association and dissociation are *K*_A_ and *K*_D_, respectively. The association and dissociation kinetic rate constants are *k*_a_ and *k*_d_, respectively. 1/*n* represents the Freundlich constant.

In selectivity studies, hemoglobin (Hb) and myoglobin (Mb), which are similar to lysozyme (Lyz, MW: 14 kDa) molecules in terms of shape, size, and molecular weight, were used as competitor agents. Hemoglobin (Hb, MW: 64 kDa) and myoglobin (Mb, MW: 17 kDa) prepared at a concentration of 50 μg/mL in phosphate buffer (pH 7.4) were first given to the system separately; then, a binary mixture of Hb+Mb and a ternary mixture of Lyz+Hb+Mb were given to the SPR system and monitored in real time. For the Lyz-AuNP-MIP sensor, it was shown that the competitor agents myoglobin and hemoglobin could not bind to the SPR sensor surface as much as lysozyme, and therefore, the SPR sensor was selective against lysozyme. AuNP-NIP kinetic analyses were also performed to demonstrate the effect of molecular imprinting. As was done in Lyz-AuNP-MIP experiments, 50 μg/mL solutions were given to the system separately, and sensor responses were observed in real time. The results showed that the binding kinetics of the AuNP-NIP sensor against competing agents and lysozyme was lower than those of the Lyz-AuNP-MIP sensors and was not even selective against lysozyme. The following equations were used to calculate the selectivity coefficient (*k*) and relative selectivity coefficients (*k*′) using values obtained from kinetic analyses for selectivity.^18^

$$k\;(\mathrm{selectivity}\;\mathrm{coefficient}):\;\Delta R_{\mathrm{template}}/\Delta R_{\mathrm{competitor}}$$ 6

$$k\prime \;(\mathrm{relative}\;\mathrm{selectivity}\;\mathrm{coefficients}):\;k_{\mathrm{MIP}}/k_{\mathrm{NIP}}$$ 7

$$\mathrm{IF}\;(\mathrm{imprinting}\;\mathrm{factor}):\;\Delta R_{\mathrm{MIP}}/\Delta R_{\mathrm{NIP}}$$ 8

In order to demonstrate the reusability of the Lyz-AuNP-MIP SPR sensor, kinetic analysis was performed by repeating four consecutive cycles of adsorption–desorption–regeneration of lysozyme solution containing 50 μg/mL using the same chip. In addition, kinetic analyses were also performed at different times such as first, second, fourth, and sixth month of reusability of the Lyz-AuNP-MIP SPR sensor. Kinetic analyses were performed with lysozyme aqueous solutions prepared at a concentration of 50 μg/mL, and the shelf life and performance of the Lyz-AuNP-MIP SPR sensor were also investigated after 6 months.

### Lysozyme Detection from Real Samples

After kinetic analyses with different solutions prepared with lysozyme solutions prepared in pH 7.4 phosphate buffer, the detection of lysozyme was also performed in artificial plasma, urine, and tear solutions. For this purpose, solutions containing 10 μg/mL lysozyme in artificial plasma, urine, and tear solutions were prepared. First, the SPR system was equilibrated by adding pH 7.4 phosphate buffer to the system for 2 min. Then, the prepared artificial plasma, urine, and tear solutions were introduced into the SPR system separately for 8 min. As a final step, the desorption solution, 10% ethylene glycol solution, was passed for 2 min, and the obtained SPR sensorgrams were recorded in real time.

## Results and Discussion

### Characterization Studies

#### Characterization of AuNPs

The AuNPs used in the preparation of SPR sensors for the determination of Lyz were characterized by transmission electron microscopy (TEM) and a Nano Zetasizer instrument (NanoS, Malvern Instruments Company, England). Based on the analysis in the TEM image, the average size of the AuNPs was determined to be 25–30 nm (Figure 2A). The homogeneous distribution and distinct shape of these gold nanoparticles indicate that control and characterization during the experimental process were successfully performed. The average size and polydispersity index (PDI) of AuNPs were measured using a Nano Zetasizer. The average size of AuNPs was measured as 47.79 nm with polydispersity index value (PdI: 0.075) (Figure 2B). AuNPs were characterized using a UV–vis spectrophotometer in the wavelength range of 200–700 nm. The highest absorbance value was observed at 450 nm for AuNPs (Figure 2C).

**Figure 2:**
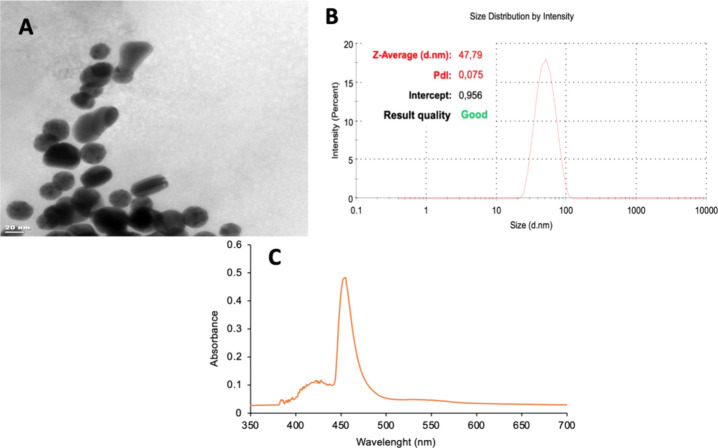
Morphological analysis and size information on the AuNPs, TEM image (A), dynamic light scattering (DLS) analysis (B), and UV–vis spectrum (C).

#### Characterization of SPR Sensors

The surface morphologies of the unmodified SPR chip and Lyz-AuNP-MIP, AuNP-NIP, and Lyz-MIP SPR sensor surfaces were characterized using atomic force microscopy (AFM) and contact angles. The contact angle of the unmodified SPR chip surface is 79.4°, while the contact angle of the Lyz-MIP SPR sensor surface is 82.9°. The contact angle increased due to the hydrophobic properties of both lysozyme and the MATrp monomer in the polymeric film on the MIP SPR sensor surface. The contact angle of the Lyz-AuNP-MIP SPR sensor surface is 78.2°, while the contact angle of the AuNP-NIP SPR sensor surface is 77.4°. The addition of hydrophilic AuNPs into the polymeric film on the SPR sensor surface caused the surface contact angles of the prepared Lyz-AuNP-MIP and AuNP-NIP SPR sensors to decrease (Figure 3).

**Figure 3:**
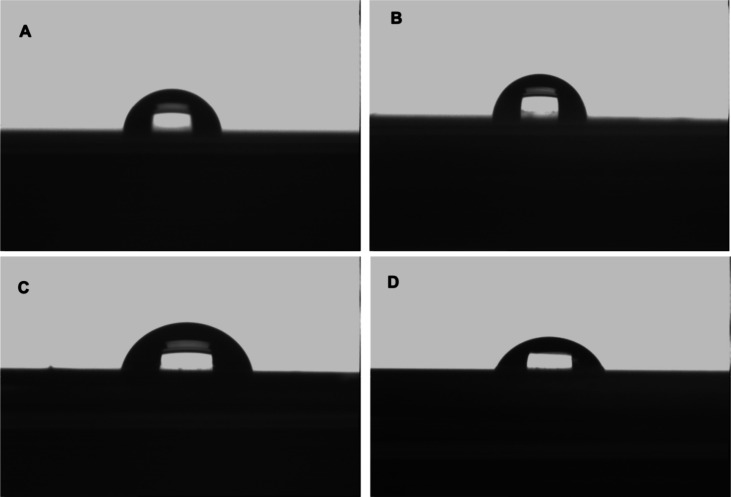
Contact angle images of (A) unmodified SPR chip and (B) Lyz-MIP, (C) Lyz-AuNP-MIP, and (D) AuNP-NIP SPR sensors.

The roughnesses of the unmodified SPR chip surface and Lyz-MIP, Lyz-AuNP-MIP, and AuNP-NIP SPR surfaces were found to be 10.50, 50.62, 78.57, and 71.32 nm, respectively. When the AFM results were examined, it was concluded that lysozyme imprinting on the SPR chip surfaces modified with allyl mercaptan was successfully imprinted (Figure 4).

**Figure 4:**
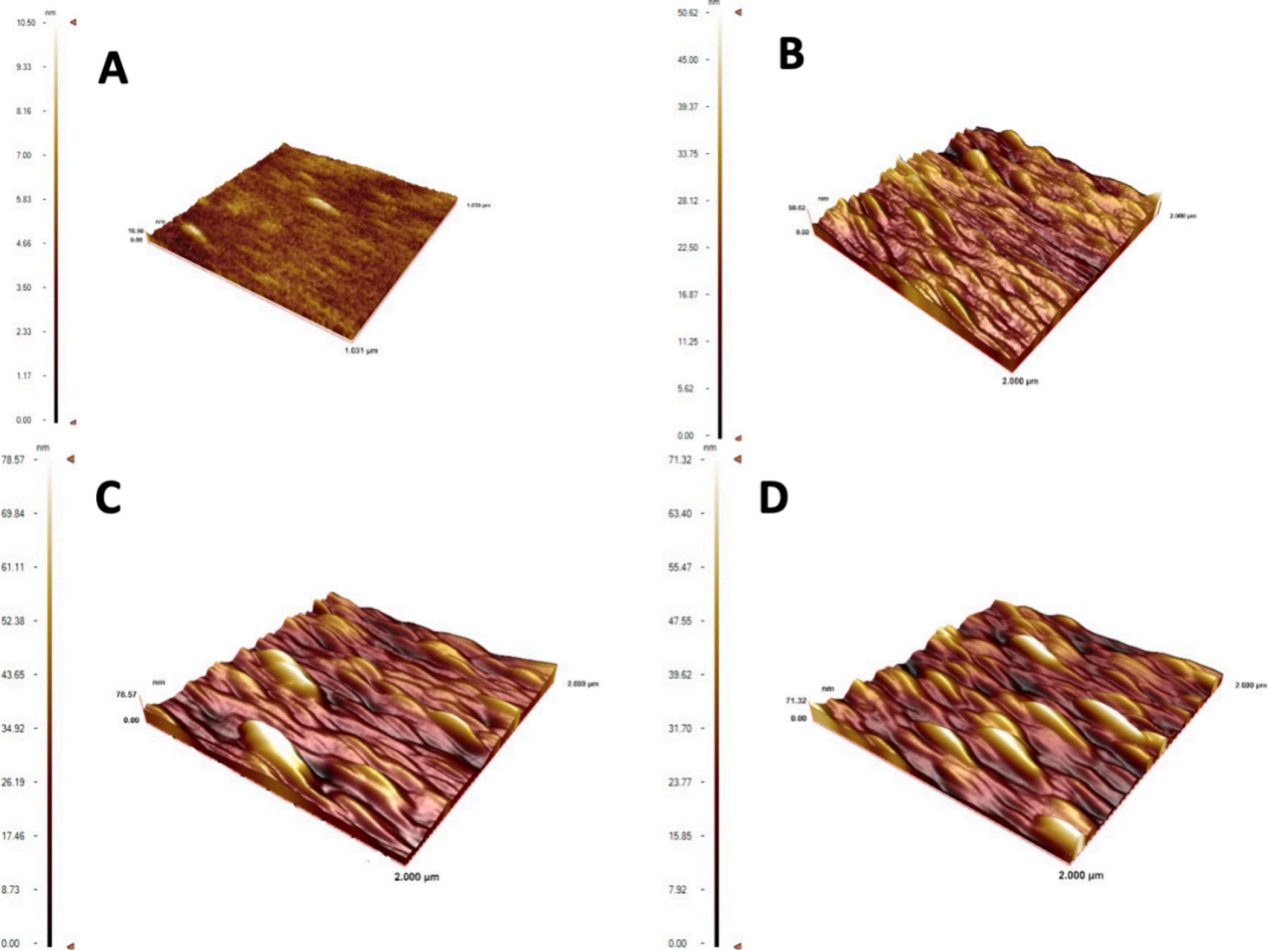
AFM images of SPR sensors: (A) unmodified SPR chip and (B) Lyz-MIP, (C) Lyz-AuNP-MIP, and (D) AuNP-NIP SPR sensors.

### Real-Time Monitoring of Lysozyme with SPR Sensors

Kinetic analyses with Lyz-AuNP-MIP, AuNP-NIP, and Lyz-MIP SPR sensors prepared for lysozyme determination were carried out using an SPRimager II device. For kinetic analyses, lysozyme aqueous solutions of 0.01–500 μg/mL concentration prepared in pH 7.4 phosphate buffer were given into the SPR system and the obtained sensorgrams were monitored in real time (Figure 5A). When the data taken in the 0.01–50 μg/mL lysozyme concentration range for the Lyz-AuNP-MIP SPR sensor are examined, the equation of the line obtained is *y* = 0.1897*x* + 0.2525 and the linearity coefficient is (*R* ^2^) 0.9855, while for the data taken in the 100–500 μg/mL lysozyme concentration range, the equation of the line obtained is *y* = 0.0061*x* + 14.115 and the linearity coefficient is (*R* ^2^) 0.9396 (Figure 5B). Using the kinetic analysis data for the Lyz-AuNP-MIP SPR sensor, the limit of detection (LOD: 3*S*/*m*) and the limit of quantification limit (LOQ: 10*S*/*m*) values were calculated. In the equation, “*S*” is the signal value (Δ*R*) when the equilibrium solution (blind solution) passes through the SPR sensor surface, while “*m*” is the slope of the calibration plot. The (Δ*R*) value for the equilibrium solution was determined as the average of the five measurements obtained, and the standard deviations of the measurements were determined as 0.0005 for the SPR sensors. Accordingly, the equation *y* = 0.1897*x* + 0.2525 for the calibration chart was used to determine the LOD as 0.008 μg/mL and the LOQ as 0.026 μg/mL.

**Figure 5:**
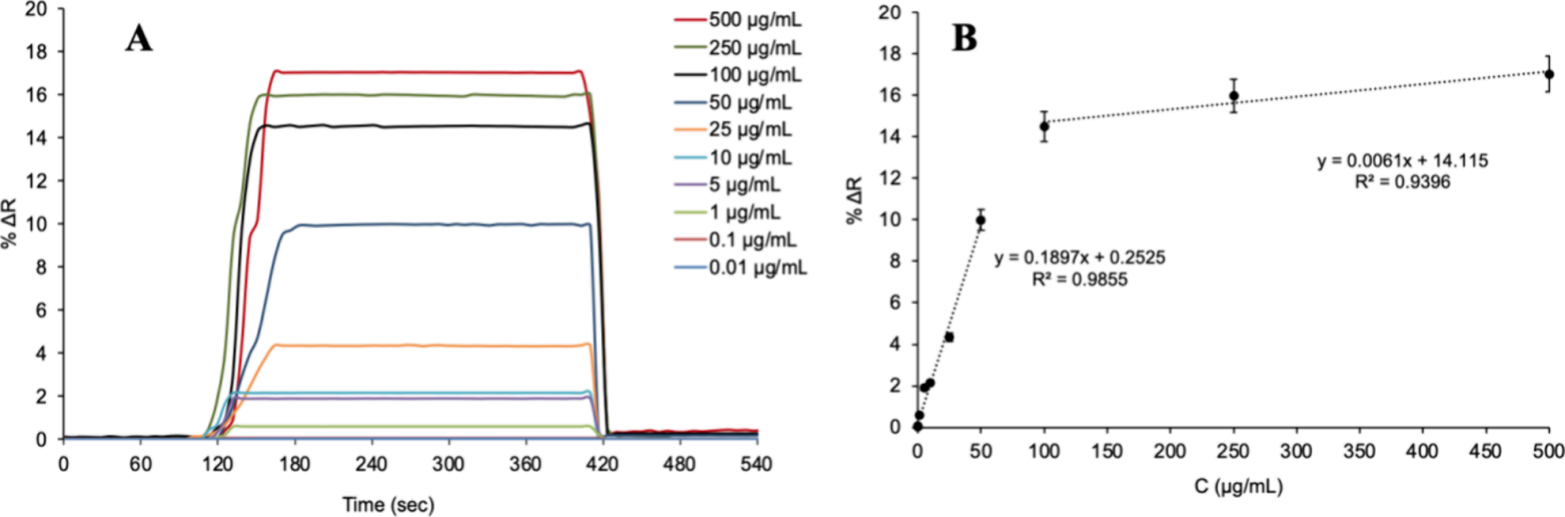
Kinetic analysis (real-time detection at different Lyz concentrations (A) and concentration dependency (B) of Lyz-AuNP-MIP SPR sensors).

For kinetic analyses using Lyz-MIP SPR sensors prepared without AuNPs, lysozyme aqueous solutions of 0.5–500 μg/mL concentration prepared in pH 7.4 phosphate buffer were given into the SPR system and the resulting sensorgrams were monitored in real time (Figure 6A). LOD and LOQ values were calculated using the kinetic analysis data in the Lyz-MIP SPR sensor. The (Δ*R*) value for the equilibrium solution was determined as 0.0015 for Lyz-MIP SPR sensors, respectively, by calculating the average of the five measurements obtained and also by the standard deviations of the measurements. Accordingly, using the equation *y* = 0.0472*x* + 0.1583 of the calibration graph, the LOD was determined as 0.095 μg/mL while LOQ was determined as 0.317 μg/mL (Figure 6B). Studies on the detection of lysozyme in the literature are shown in Table 1 below.

**Figure 6:**
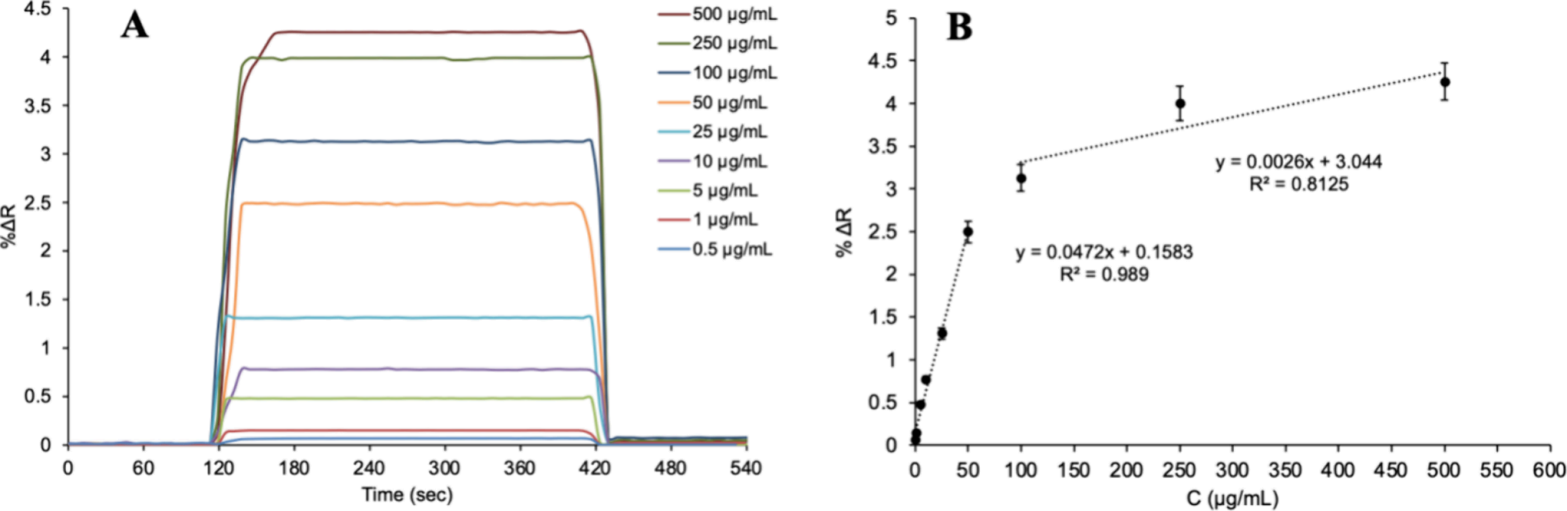
Kinetic analysis (the real-time detection at different Lyz concentrations (A) and concentration dependency (B) of Lyz-MIP SPR sensors).

**Table 1:** Brief Summary of Different Sensor Studies for Lysozyme Detection in the Literature

type of sensor	range of kinetic studies	limit detection	sample	surface modification	refs.
electrochemical	20 μM to 150 nM	62 and 141 nM	artificial saliva	polyscopoletin film	^19^
SPR	1–500 nM	0.66 nM	egg white	PMAPA NP	^20^
SPR	0–40 μg/mL	3.5 nM	undiluted serum	rGO	^21^
L-SPR	3–150 nM	1.5 nM	milk	AgNP	^22^
SPR	21–1400 nM	0.084 nM	egg white	PEDMAH NP	^23^
SPR	0.05–80 μg/mL	2.4 nM	wine	aptamer	^24^
FRET	0–1.75 μM	85 nM	human serum samples	pAuNPs	^25^
fluorescence sensor	1 × 10^–5^ to 1 × 10^–6^ M	2.86 × 10^–7^ and 4.76 × 10^–7^ M	artificial blood serum	silver nanocluster	^26^
FRET	0–5 μM	30 nM	egg white	aptamer	^27^
fluorescence sensor	0.2–2.0 μM	4.53 × 10^–3^ μM	human urine and egg white	MIP@MNP/QDs	^28^
fluorescence sensor	10 pM to 2.0 nM	3.6 pM	human serum	+AuNRs and DNA/AgNCs	^29^
fluorescence sensor	0.03–10 μM	7 nM	human urine	dNIR-CDs	^30^
crystal sensor	0–1.0 mg/mL	1.38 × 10^–3^ mg/mL	artificial tears and human urine	2D colloidal crystal hydrogel	^31^
fluorescence sensor	10–120 μg/mL	3.2 μg/mL	human serum and egg white	MIP@CdTe QDs	^32^
SPR sensor	0.01–500 μg/mL	0.008 μg/mL	artificial plasma	AuNP	in this study
			artificial tear		
			artificial urine		

Association and binding kinetic analyses and different isotherm models were investigated using data obtained from kinetic analyses performed with the Lyz-AuNP-MIP SPR sensor at different lysozyme concentrations (Figures S1 and S2). According to the kinetic analysis results, binding kinetic analysis showed that lysozyme binds to the Lyz-AuNP-MIP sensor surface with 99% accuracy. Furthermore, it was observed that the most suitable model for the determination of lysozyme fits the Langmuir adsorption isotherm model. This indicates that the initial lysozyme binding to the Lyz-AuNP-MIP SPR sensor is homogeneous, and the monolayer has minimal lateral interaction. According to the constants obtained, the theoretical Δ*R*_max_ value calculated in the Langmuir adsorption isotherm model (19.08) was very close to the experimental Δ*R*_max_ value (17.02). Also, *R*_max_, *R* ^2^, *K*_A_, and *K*_D_ values obtained from the lines are given in Table S1.

### Selectivity of SPR Sensors

In selectivity studies, myoglobin (Mb, MW: 17 kDa) and hemoglobin (Hb, MW: 64.5 kDa), which are similar to lysozyme molecules in terms of shape, size, and molecular weight, were used as competitive agents. The prepared myoglobin and hemoglobin solutions at a concentration of 50 μg/mL in phosphate buffer (pH 7.4) were first given to the SPR system separately. Then, the binary Mb+Hb mixture and the triple Lyz+Mb+Hb mixture were given to the SPR system, and kinetic analyses were performed and sensorgrams were recorded. As in every kinetic analysis, the analyses were completed by passing 1.0 M pH 7.4 phosphate buffer through the Lyz-AuNP-MIP SPR sensor surface at a flow rate of 2 mL/min for 2 min, followed by 5 min of adsorption solution, and finally 2 min of (10%) ethylene glycol desorption solution through the SPR system. For the Lyz-AuNP-MIP sensor, it was shown that the competitive agents myoglobin and hemoglobin could not bind to the SPR sensor surface as much as lysozyme, and the SPR sensor was selective against lysozyme. As Lyz-AuNP-MIP SPR sensor’s kinetic analysis experiments were performed, all solutions prepared in the change of 50 μg/mL by using the AuNP-NIP SPR sensor were given separately and the sensor responses were observed in real time (Figure 7).

**Figure 7:**
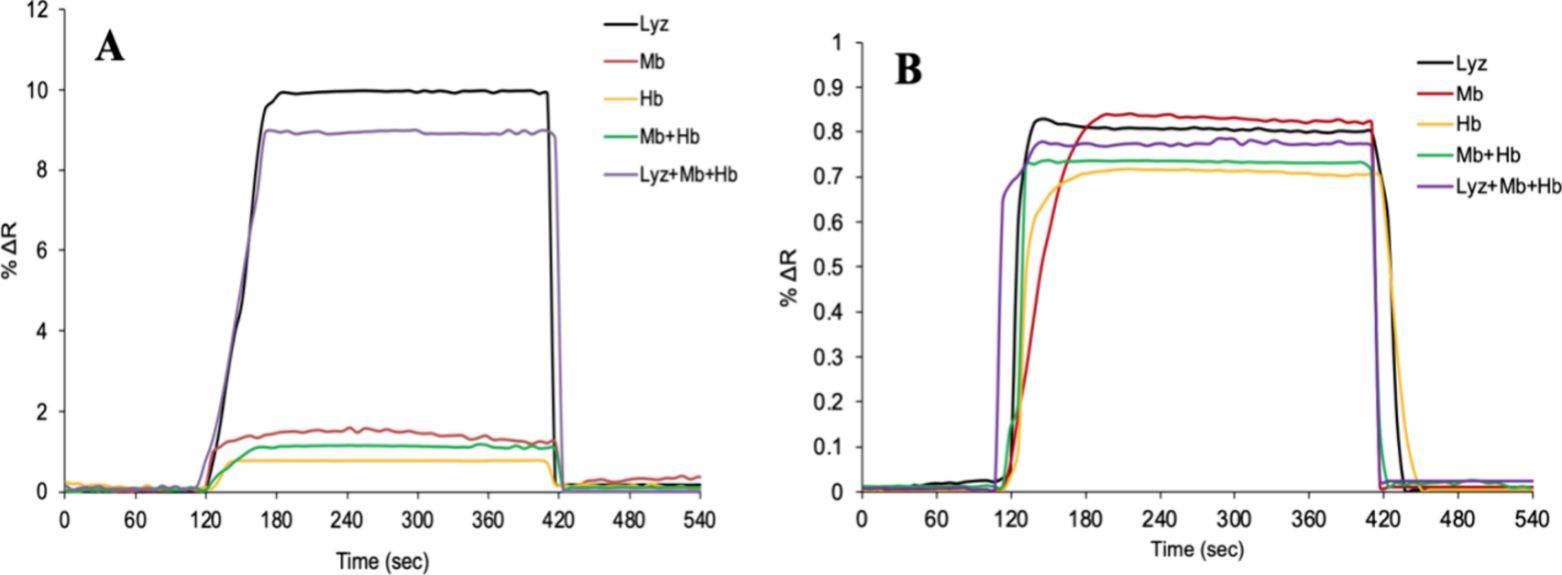
Selectivity sensorgrams of Lyz-AuNP-MIP (A) and AuNP-NIP (B) SPR sensors.

When the kinetic analyses with Lyz-AuNP-MIP and AuNP-NIP SPR sensors were compared, it was observed that the SPR signal for the lysozyme molecule decreased from 9.94 to 0.81. The relative selectivity coefficients (*k*′) of the Lyz-AuNP-MIP SPR sensor for Lyz/Mb and Lyz/Hb are 6.88 and 11.18, respectively (Table 2). In addition, the selectivity of the Lyz-AuNP-MIP sensor was calculated by the imprinting factor (IF) determined as Δ*R*_MIP_/Δ*R*_NIP_. The imprinting factor of the Lyz-AuNP-MIP sensor was calculated as 12.27. According to all selectivity analyses, it shows that the imprinting factor for lysozyme is higher than other competitive agents. These results show that the prepared SRP by the molecular imprinting method significantly increases the adsorption selectivity of the polymeric film on the sensor surface and that the specific recognition sites on the sensor surface are not suitable for other competitive agents.

**Table 2:** Selectivity Parameters of Lyz-AuNP-MIP and AuNP-MIP SPR Sensors

	Lyz-AuNP-MIP SPR sensor		AuNP-NIP SPR sensor		
molecule names	Δ*R*	*k*	Δ*R*	*k*	*k*′
Lyz	9.94		0.81		
Mb	1.49	6.67	0.83	0.97	6.88
Hb	0.78	12.74	0.71	1.14	11.18
Mb+Hb	1.15	8.64	0.73	1.11	7.78
Lyz+Mb+Hb	8.98	1.11	0.79	1.02	1.08

### Clinical Analyses

Kinetic analyses for the determination of lysozyme in artificial plasma, artificial urine, and artificial tear samples were investigated using Lyz-AuNP-MIP SPR sensors. For this purpose, artificial plasma, artificial urine, and artificial tear solutions containing 10 μg/mL lysozyme were prepared. The kinetic analysis was performed by preparing an artificial plasma solution added at a concentration of 10 μg/mL. First, pH 7.4 phosphate buffer was added to the SPR system for 2 min and artificial plasma solution to which lysozyme was added for 5 min, and as a final step, 10% ethylene glycol solution, which is a desorption solution, was given from the system for 2 min and kinetic analyses were performed in real time. All kinetic analysis steps for the determination of lysozyme in artificial plasma were performed with lysozyme solutions prepared in artificial urine and artificial tear (Figure S3). The obtained recoveries from lysozyme solutions in artificial plasma, artificial urine, and artificial tear solutions were calculated as 99.78, 99.71, and 99.89, respectively.

### Reusability of SPR Sensors

To test the reusability of the Lyz-AuNP-MIP SPR sensor, lysozyme solution at a concentration of 50 μg/mL was introduced into the SPR system four times consecutively (Figure 8A). As described in the kinetic analysis section, experiments were performed by first passing through the SPR system with pH 7.4 phosphate buffer for 2 min, then with 50 μg/mL containing lysozyme solution for 5 min, and finally with 10% ethylene glycol desorption solution for 2 min. No significant decrease in the signal level of the Lyz-AuNP-MIP sensor was observed after four cycles. The percentage efficiency of the SPR sensor was calculated to be 98.6. The reusability of the Lyz-AuNP-MIP SPR sensor was tested in different months, and kinetic analysis was performed with samples prepared using aqueous solutions containing 50 μg/mL lysozyme (Figure 8B). The efficiency of the Lyz-AuNP-MIP SPR sensor was 89%. These findings reveal that Lyz-AuNP-MIP SPR sensors exhibit high kinetic efficiency and stability in terms of reusability at different times. This property is important for evaluating the long-term performance of the sensor and its reliable use in various applications. The high efficiency ratio emphasizes that the sensor exhibits a strong and stable performance, in terms of reusability. These results support the potential for the continued and effective use of Lyz-AuNP-MIP SPR sensors in practical applications.

**Figure 8:**
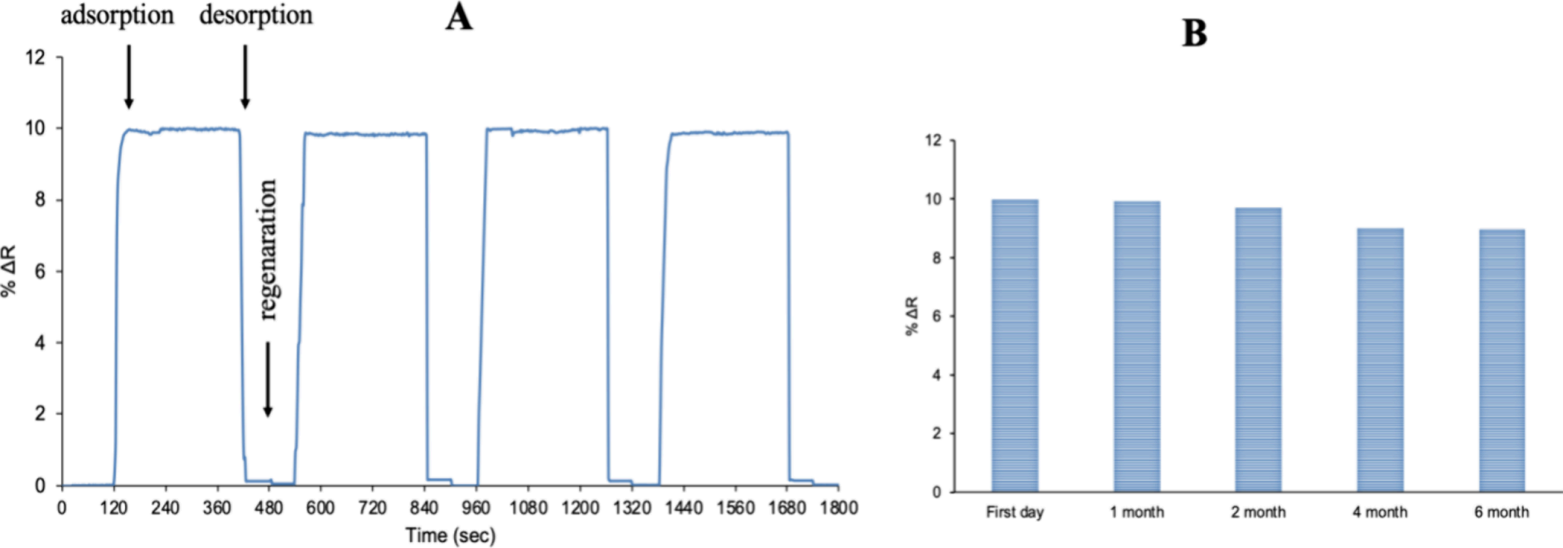
Reusability of Lyz-AuNP-MIP SPR sensors on the same day (A) and different days (B).

## Conclusions

Lysozyme, which is important in increasing the body’s resistance, is of great importance in the diagnosis of many diseases such as infection, cold, bronchitis, asthma, leukemia, cancer, Crohn’s disease, and rheumatoid arthritis.^33^ In this study, Lyz-AuNP-MIP and AuNP-NIP SPR sensors containing lysozyme-specific recognition sites were prepared for real-time determination of the lysozyme. AuNPs were used to reduce the detection limit of lysozyme and increase the sensitivity of the SPR signal response. A LyZ-MIP SPR sensor without AuNPs was also prepared to study the effect of incorporation of AuNPs used to enhance the SPR signal response. After surface characterization of the prepared Lyz-AuNP-MIP SPR sensors by AFM and contact angle, kinetic analysis for the determination of lysozyme showed that lysozyme can be detected in real time with a correlation coefficient of 0.9855 for the lysozyme concentration range of 0.01–500 μg/mL. Furthermore, the selectivity of the Lyz-AuNP-MIP SPR sensor for lysozyme was found to be 6.88 and 11.18 times more selective than those for myoglobin and hemoglobin molecules, respectively. It was also concluded that the Lyz-AuNP-MIP SPR sensor was able to detect lysozyme with no appreciable decrease in performance in four consecutive reusability analyses. Lysozyme was detected using the Lyz-AuNP-MIP SPR sensor not only in lysozyme solutions prepared in pH 7.4 phosphate buffer but also in complex media such as artificial plasma, artificial urine, and artificial tear to evaluate the matrix effect, and recoveries were found to be about 97%. Finally, it can be concluded that the Lyz-AuNP-MIP SPR sensor prepared using a molecular imprinting method can be used in clinical applications and is a good proof of concept for the future and further development of SPR sensors.

## Supplementary Material



## Notes

Results of association and binding kinetic analyses, results of adsorption isotherm parameters, and real-time sensorgrams for lysozyme determination in complex samples such as artificial plasma, urine, and tears of the gold nanoparticle-modified lysozyme-imprinted (Lyz-AuNP-MIP) surface plasmon resonance sensor (PDF)
