# Supplementary material for: Lysozyme-Imprinted Surface Plasmon Resonance Chips Decorated with Gold Nanoparticles for Lysozyme Detection
Source: ACS Omega. 2025 Jun 26;10(26):28055–64. doi: 10.1021/acsomega.5c01607 (PMC12242623; doi:10.1021/acsomega.5c01607)
Supplement: Supplementary file 1 [file ao5c01607_si_001.pdf]

Lysozyme Imprinted Surface Plasmon Resonance Chips Decorated with Gold Nanoparticles for Lysozyme Detection

Şeyma Eriş[a], Duygu Çimen[b], Adil Denizli[b*]
[a]Hacettepe University, Department of Chemistry, Ankara, Turkey
[b]Hacettepe University, Bioengineering Division, Ankara, Turkey

Association and binding kinetic analyses and different isotherm models were investigated using data obtained from kinetic analyses performed with Lyz-AuNP-MIP SPR sensor at different lysozyme concentrations.

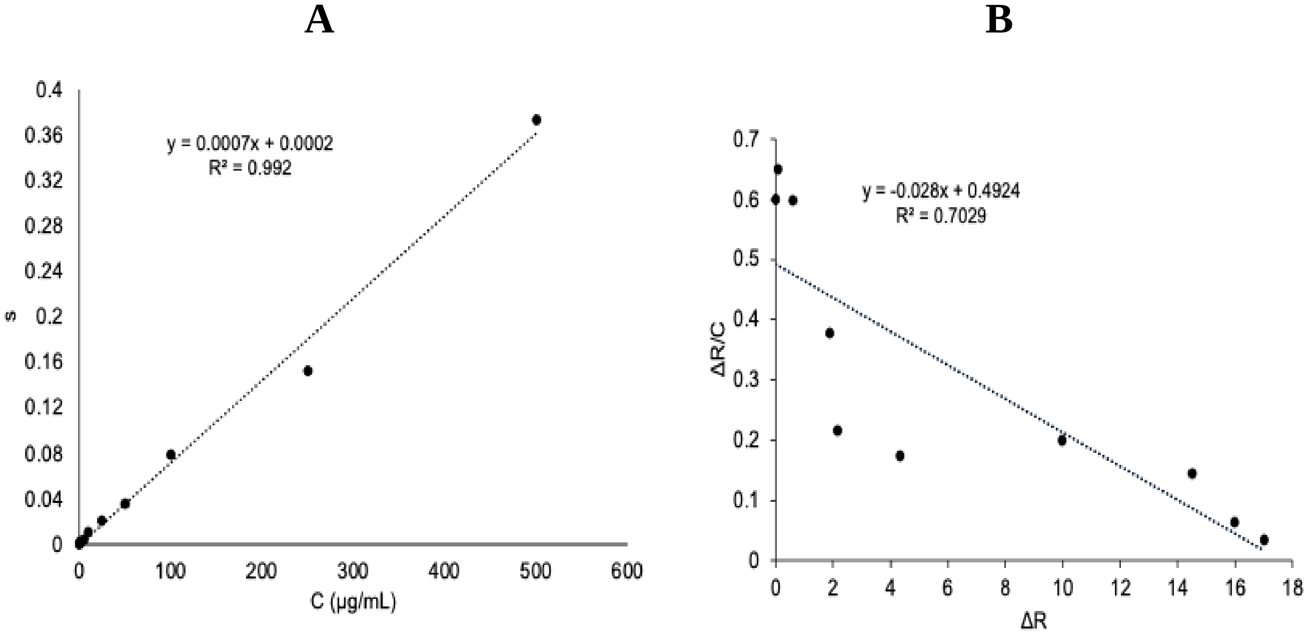

Figure S1: Determination of the kinetic binding constants between Lyz and Lyz-AuNP-MIP SPR sensor (association kinetics analysis (A), and equilibrium analysis (Scatchard) (B)).

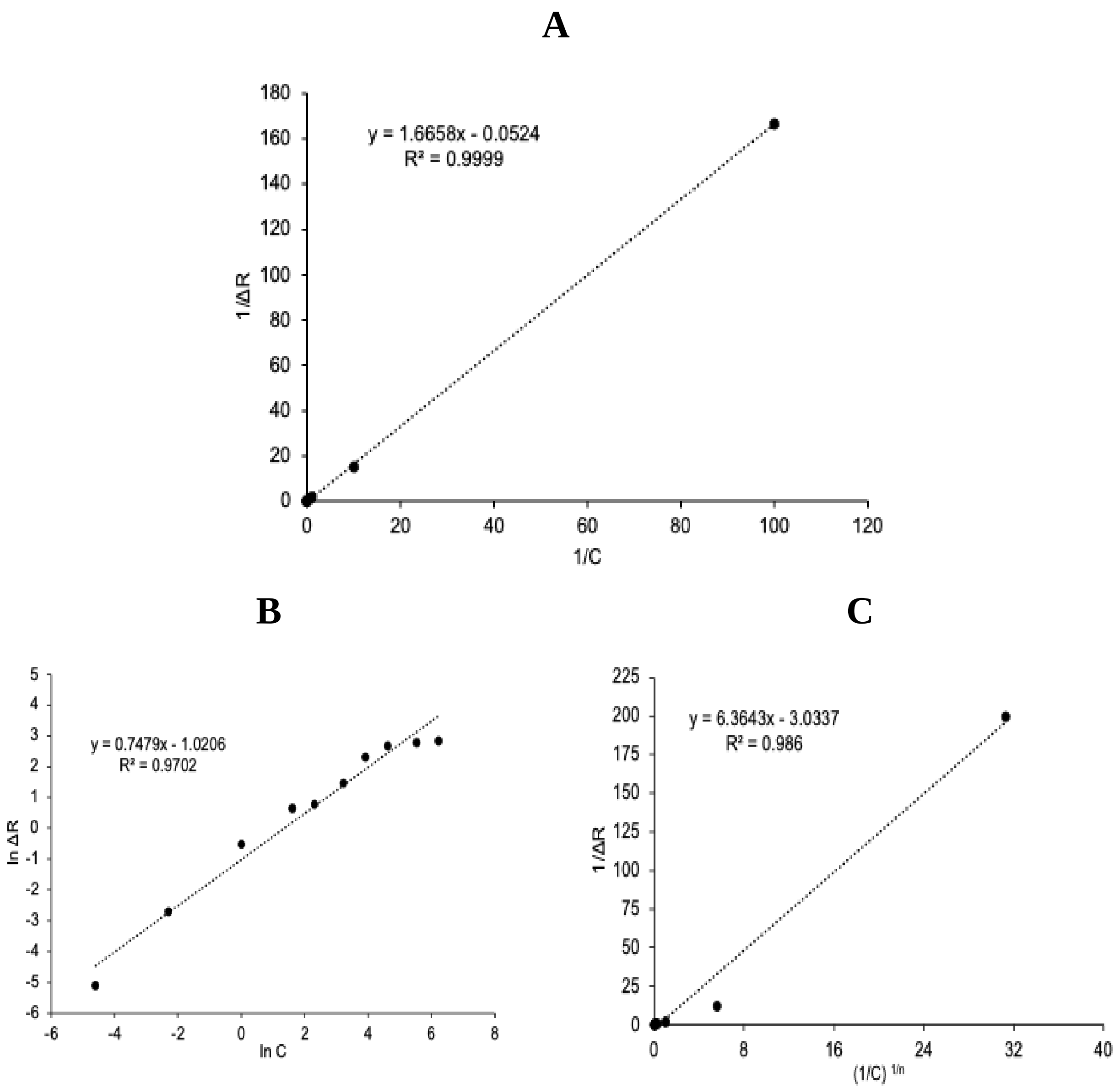

Figure S2: Kinetic Isotherm Models (Langmuir (A), Freundlich (B), and Langmuir-Freundlich (C).

Table S1. Kinetic and isotherm parameters of Lyz-AuNP-MIP SPR sensor.

**Equilibrium analysis (Scathard)**	**Association kinetics analysis**	**Langmuir**	**Freundlich**	**Langmuir-Freundlich**
ΔR_{max}: 17.59	k_a, (µg/mL)$^{-1}$.s^{-1}: 0.0007	$\Delta R_{max:}$ 19.08	ΔR_{max}: 2.77	ΔR_{max}: 0.329
K_A, (µg/mL)$^{-1}$:0.028	k_d, s^{-1}: 0.0002	K_A, (µg/mL)$^{-1}$. 0.031	1/n: 0.7479	K_A, (µg/mL)$^{-1}$: 0.476
K_D, µg/mL: 35.71	K_A, (µg/mL)$^{-1}$: 3.5	K_D, µg/mL: 31.79	R^2: 0.9702	K_D, µg/mL: 2.09
R^2: 0.7029	K_D, µg/mL: 0.285	R^2: 0.999		1/n: 0.7479
	R^2: 0.992			R^2: 0.986

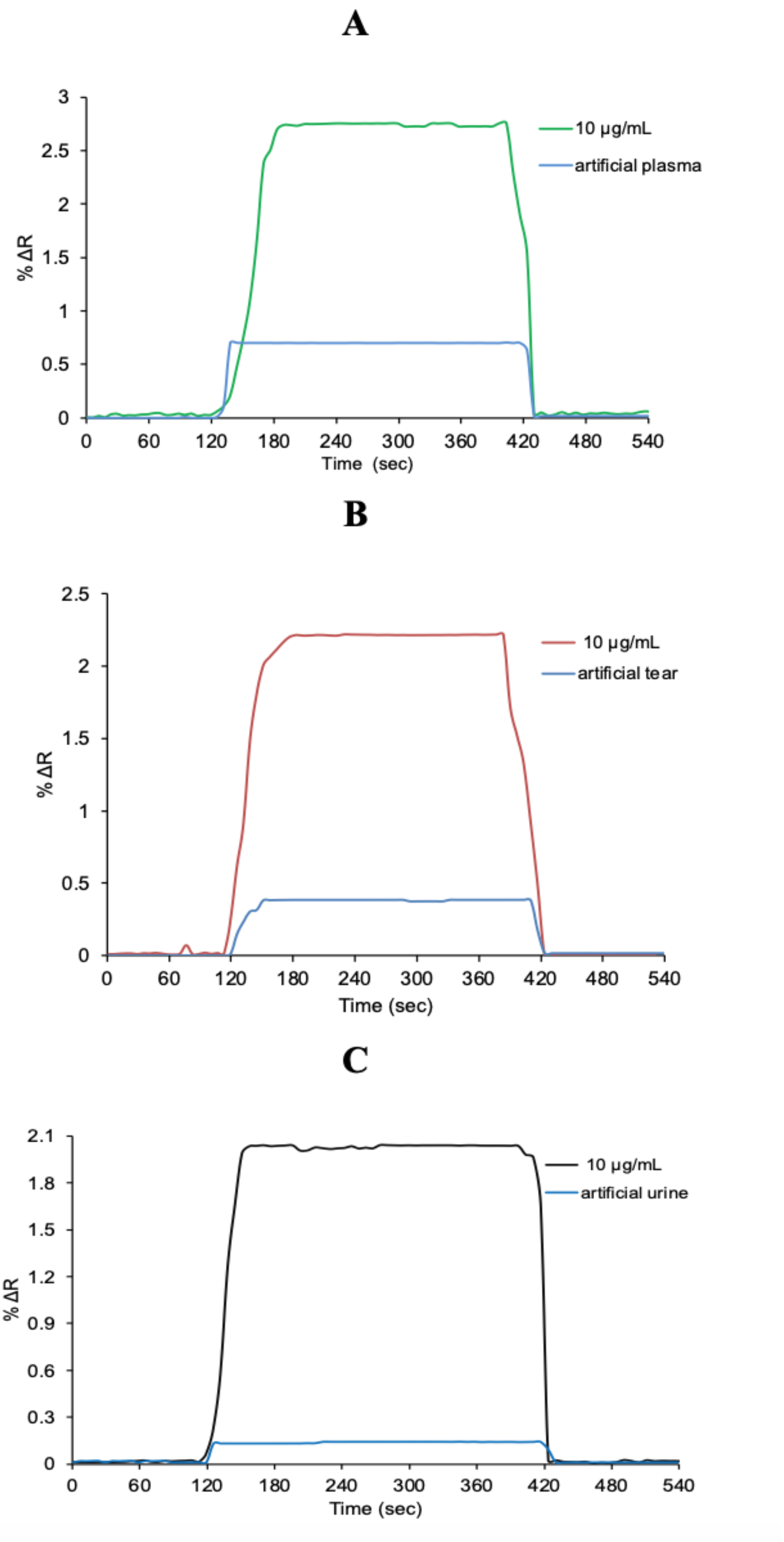

Figure S3. Real-time sensorgrams for lysozyme detection from artificial plasma (A), artificial tear (B), and artificial urine (C) samples with Lyz-AuNP-MIP SPR sensors.
